# The current status, challenges, and future perspectives for managing diseases of brassicas

**DOI:** 10.3389/fmicb.2023.1209258

**Published:** 2023-07-18

**Authors:** Shannon F. Greer, Arthy Surendran, Murray Grant, Robert Lillywhite

**Affiliations:** ^1^School of Life Sciences, University of Warwick, Coventry, United Kingdom; ^2^Carbon, Crop and Soils Group, SRUC, Edinburgh, United Kingdom

**Keywords:** *Brassica oleracea*, integrated pest management, black rot, clubroot, downy mildew, turnip yellows virus, aphids, plant disease

## Abstract

The *Brassica* genus comprises the greatest diversity of agriculturally important crops. Several species from this genus are grown as vegetable and oil crops for food, animal feed and industrial purposes. In particular, *B. oleracea* has been extensively bred to give rise to several familiar vegetables (cabbage, broccoli, cauliflower, kale and Brussels Sprouts, etc.) that are grouped under seven major cultivars. In 2020, 96.4 million tonnes of vegetable brassicas were produced globally with a 10.6% increase over the past decade. Yet, like other crops, the production of brassicas is challenged by diseases among which, black rot, clubroot, downy mildew and turnip yellows virus have been identified by growers as the most damaging to UK production. In some cases, yield losses can reach 90% depending upon the geographic location of cultivation. This review aims to provide an overview of the key diseases of brassicas and their management practices, with respect to the biology and lifecycle of the causal pathogens. In addition, the existing controls on the market as well as those that are currently in the research and development phases were critically reviewed. There is not one specific control method that is effective against all the diseases. Generally, cultural practices prevent disease rather than reduce or eliminate disease. Chemical controls are limited, have broad-spectrum activity, are damaging to the environment and are rapidly becoming ineffective due to the evolution of resistance mechanisms by the pathogens. It is therefore important to develop integrated pest management (IPM) strategies that are tailored to geographic locations. Several knowledge gaps have been identified and listed in this review along with the future recommendations to control these four major diseases of brassicas. As such, this review paper will act as a guide to sustainably tackle pre-harvest diseases in *Brassica* crops to reduce food loss.

## 1. Introduction

**Figure 1:**
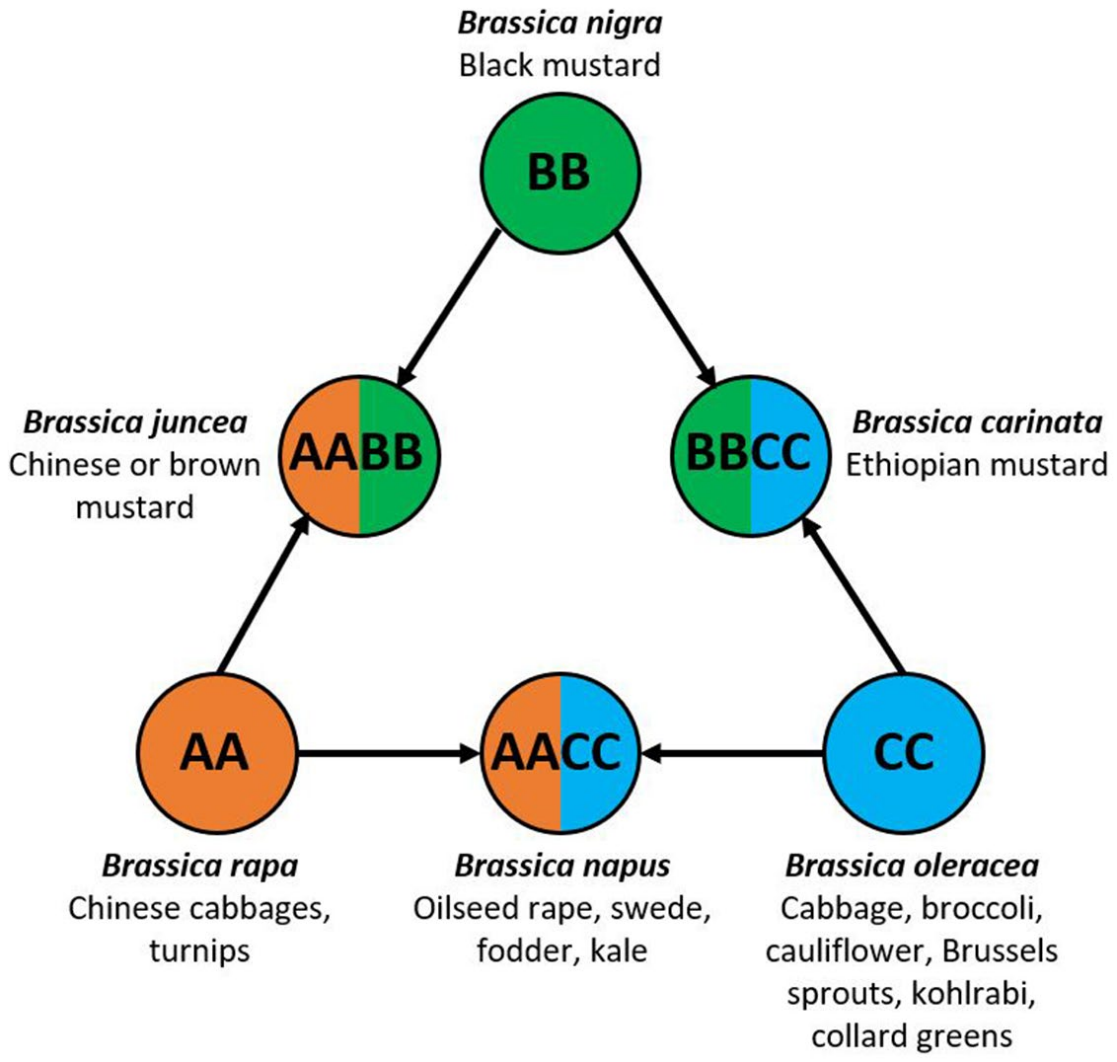
The triangle of ‘U’ showing the genomic relationships of six agronomically important *Brassica* species and their commonly associated crop types. Modified from Nagaharu (1935).

*Brassica* is a genus in the *Brassicaceae* family, more colloquially known as the mustard or cabbage family. There are 37 species of *Brassica* (Gupta, 2016), many of which are of agricultural, economical and societal importance. Brassicas are the oldest and most widely consumed plants worldwide and are highly nutritious (Jabeen, 2020). They are consumed as vegetables, oils and condiments that are rich in vitamins A and C, antioxidants, dietary fiber, calcium, magnesium, and iron. The genomic relationship of the six most cultivated *Brassica* species is often depicted by the Triangle of ‘U’ (Figure 1; Nagaharu, 1935). The corner diploid species are *Brassica rapa* (genome AA, Chinese cabbage and turnips), *Brassica nigra* (genome BB, black mustard) and *Brassica oleracea* (genome CC, vegetable brassicas). The hybridisation of the diploid genomes gave rise to the allotetraploid species *Brassica juncea* (genome AABB, Chinese or brown mustard), *Brassica napus* (genome AACC, oilseed rape, swede and fodder kale) and *Brassica carinata* (genome BBCC, Ethiopian mustard).

**Figure 2:**
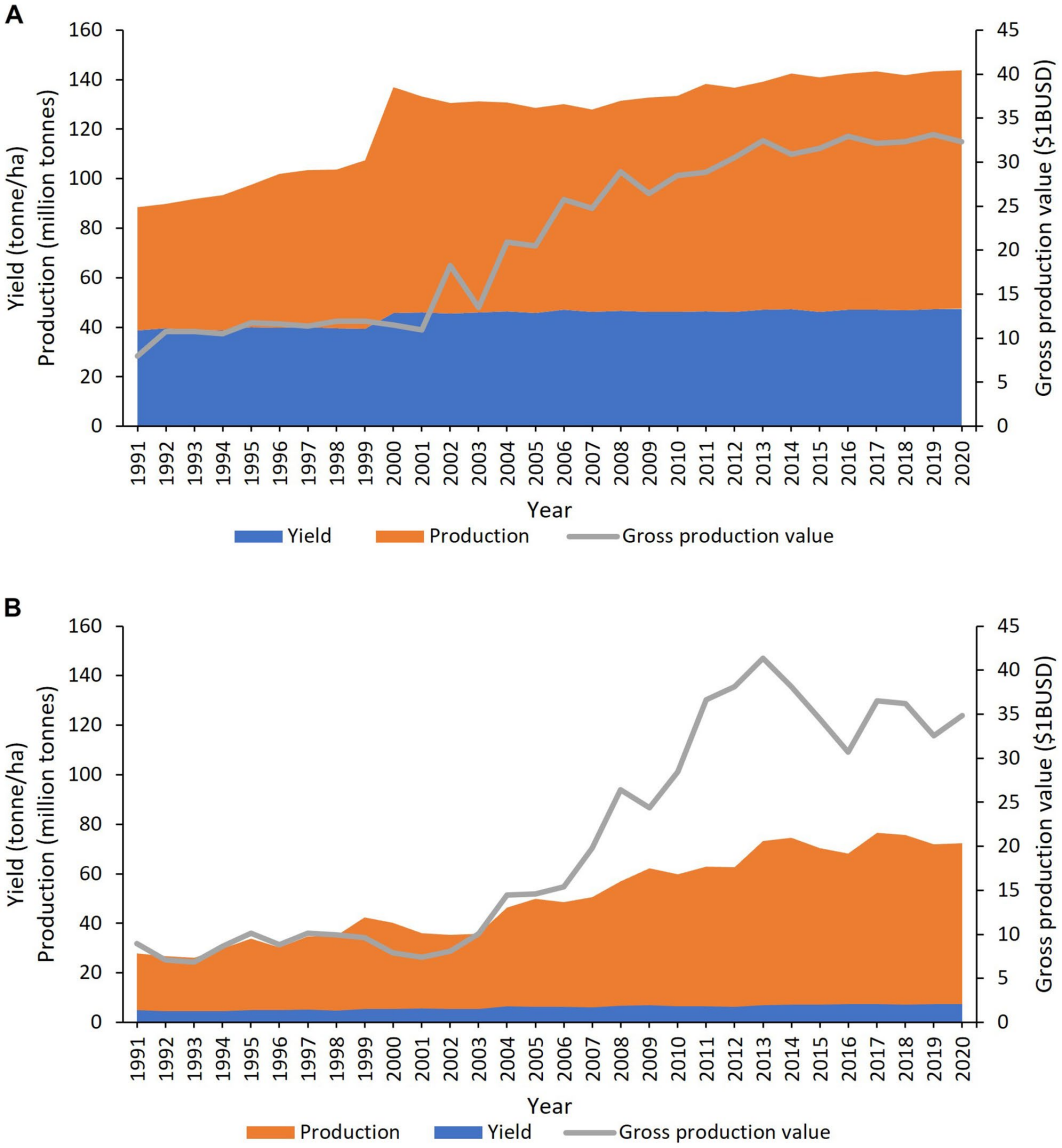
Worldwide production (orange), yield (blue) and gross production value (grey) of **(A)** vegetable brassicas (*Brassica oleracea*) cabbage, cauliflower and broccoli. **(B)** Oilseed brassicas (*Brassica napus*). Data from FAO (2023a,c).

Of the six species in the triangle of U, *B. oleracea* is the most diverse and extensively bred. Within the species there are several subspecies/varieties, *acephela* (kale and collard greens), *alboglabra* (Chinese kale and broccoli), *botrytis* (cauliflower and Romanesco broccoli), *capitata* (cabbage), *gemmifera* (Brussels sprouts), *gongylodes* (kohlrabi) and *italica* (broccoli) (Labana and Gupta, 1993; Figure 1). Collectively they are known as the vegetable brassicas, and they grow best in temperate regions but are grown in most countries worldwide. The turn of the millennium saw a massive 33.8% increase in the production of vegetable brassicas like cabbage, cauliflower and broccoli from 68.1 to 91.1 million tonnes but since then, production has remained constant at an average of 89.7 million tonnes *per annum* (yearly average 2000–2020) (FAO, 2023a; Figure 2A). The largest producers of cabbage in 2020 were China (35.0 million tonnes), India (9.3 million tonnes) and Russia (2.6 million tonnes) (FAO, 2023a). With improvements in production and yield, the gross value of cabbage, cauliflower and broccoli has tripled to $32.3 billion USD in 2020 (FAO, 2023c; Figure 2A), underlining their global importance in both nutritional and food security.

The oilseed brassicas comprise of *B. napus* and *B. juncea* and less significantly *B. carinata* and *B. rapa*. Each have their vegetable forms. Rapeseed oil is the third most important oil after palm (*Elaeis guineensis*) and soybean (*Glycine max*) (USDA, 2020) and depending on the oil composition are used as edible oils, industrial lubricant and biofuels. The seed meal produced as a by-product of oil production, is used as a nutrient rich animal feed. From 2000 to 2020 rapeseed production increased 79.9% from 40.2 to 72.3 million tonnes but yield has remained consistently low at 1.8 tonnes/ha (FAO, 2023a; Figure 2B). In 2020, the largest producers of rapeseed were Canada (19.5 million tonnes), China (14.0 million tonnes) and India (9.1 million tonnes) (FAO, 2023a). Like vegetable brassicas, oilseed brassicas had a similar production value of $34.9 billion USD in 2020 (Figure 2B).

The FAO state that under ideal conditions cabbages could yield as much as 77 tonnes/ha but realized yield has remained consistently lower, averaging 46.5 tonnes/ha *per annum* (FAO, 2023a,b; Figure 2A). Similarly, rapeseed is only reaching a fifth of its predicted 9.2 tonnes/ha maximum yield potential (Berry and Spink, 2006; Figure 2B). Several biotic and abiotic factors contribute to this gap in achieved and potential yield, most notably pests and diseases which are thought to cause crop losses of 20–40% worldwide (Savary et al., 2012). The UK’s Research and Innovation strategic guidance, through consultation with farmers and growers has identified black rot (*Xanthomonas campestris* pv. *campestris*), aphids and vectored viruses (turnip yellows virus), downy mildew (*Hyaloperonospora parasitica*) and clubroot (*Plasmodiophora brassicae*) as the major pests and diseases of brassicas (UKRI, 2022). In agriculture, plant protection is delivered by one or more combinations of the following methods farm practise, chemical and biological controls, resistance cultivars and integration of all these methods, known as an integrated pest management (IPM) strategy. In this review, we will discuss the current knowledge of these diseases and their management practices with respect to the biology and lifecycle of the causal pathogens. We will critically review the existing controls on the market and those in the research and development phases. For each disease we have provided existing and emerging control strategies and also highlighted the current knowledge gaps.

## 2. Black rot

### 2.1. Host and impact

Black rot of the *Brassicaceae* family is caused by the Gram-negative bacterium *Xanthomonas campestris* pv. *campestris* (*Xcc*). The disease was first reported in Kentucky, USA in 1889 (Garman, 1890) and the casual pathogen later described in 1895 (Pammel, 1895), the name of which has changed several times since, *Bacillus campestris* (Pammel, 1895), *Pseudomonas campestris* (Smith, 1898), *Bacterium campestre* (Smith, 1898), *Phytomonas campestris* (Bergey et al., 1923) and *Xanthomonas campestris* pv. *campestris* (Dowson, 1939). There are nine races of *Xcc*, which are defined by their pathogenicity on a panel of *Brassica* accessions (Vicente et al., 2001). Races 1, 4, 5, and 6 are the most prevalent worldwide, with races 1 and 4 most common in vegetable brassicas (Vicente et al., 2001; Jensen et al., 2010; Mulema et al., 2012; Singh et al., 2016).

**Figure 3:**
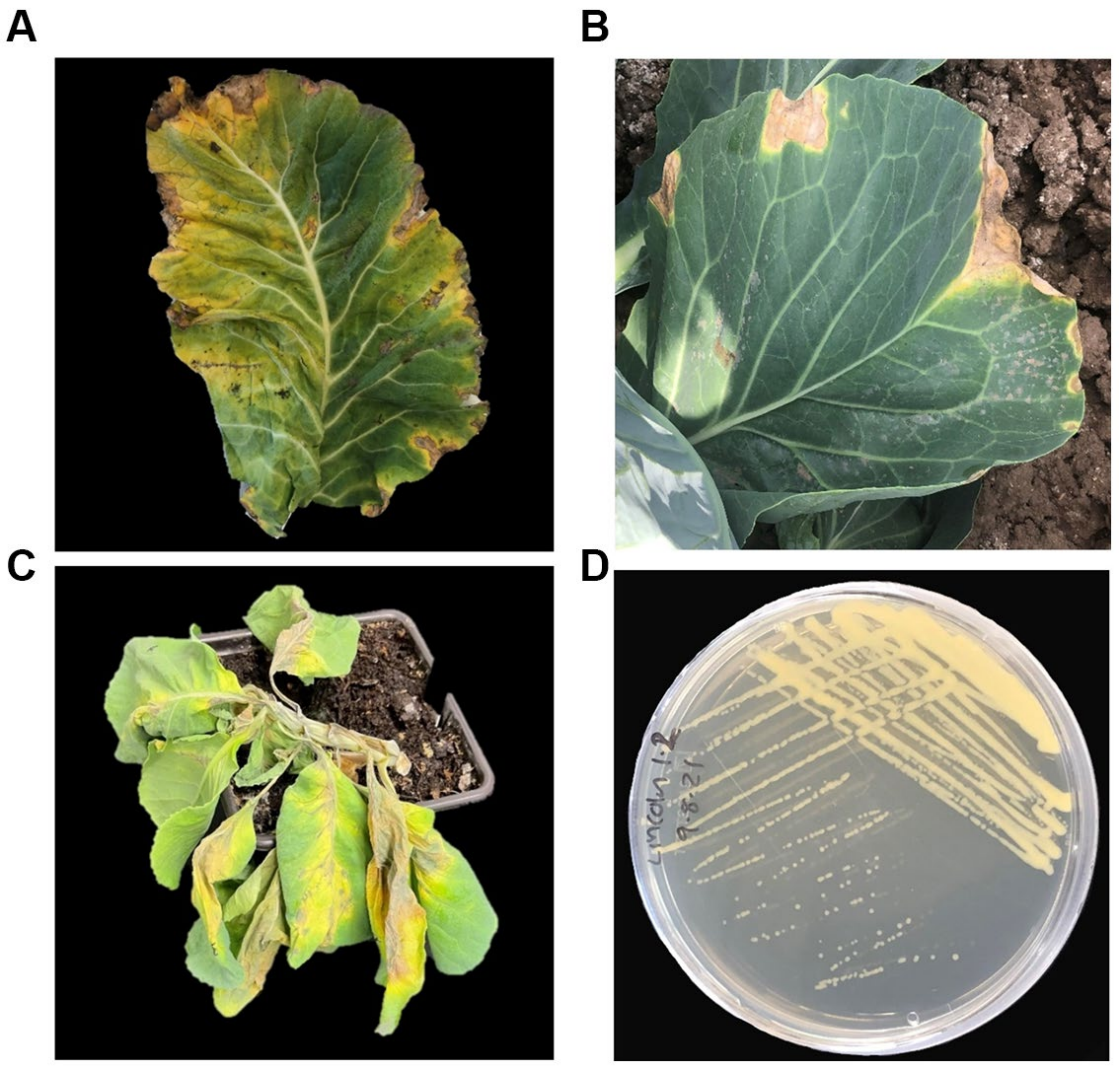
Disease symptoms and morphology of *Xanthomonas campestris* pv. *campestris* (*Xcc*). Symptoms of black rot caused by *Xcc* on **(A)** cauliflower (*Brassica oleracea* var. *botrytis*) and **(B)** cabbage (*Brassica oleracea* var. *botrytis*). **(C)** Wilt of cabbage caused by *Xcc* (photograph by Srayan Ghosh). **(D)** *Xcc* plated on King’s B media.

Black rot symptoms are distinct, characterized by ‘V’ shaped chlorotic and necrotic lesions (Figure 3) that begin at the leaf margin, where *Xcc* enters the vascular xylem tissue through hydathodes. The lesions expand as *Xcc* migrates toward the leaf mid vein and the vascular tissue can blacken. In severe cases, *Xcc* can move systemically throughout the plant causing wilting and rot (Figure 3C).

*Xcc* is now present in 93 countries and 6 continents (CABI, 2022d). It is considered the most damaging bacterial disease of vegetable brassicas worldwide, causing yield losses of up to 60% in susceptible cultivars (Gupta, 1991; McKeen, 2009). *Xcc* also infects oilseed brassicas but is not widely reported and its impact on yield and oil quality is yet to be investigated (Popović et al., 2013, 2019). *Xcc* can act as a gateway pathogen for other diseases like soft rots caused by *Pseudomonas* and *Pectobacterium* species that can lead to total losses of stored brassicas like white cabbage. *Xcc* thrives in warm, humid conditions and so disease outbreaks could become more frequent and yield losses exacerbated with climate change (Kůdela et al., 2018).

### 2.2. Pathogen lifestyle and transmission

*Xcc* is a seed-transmissible disease, and its worldwide distribution is primarily the result of trading and sowing infected seed. This is supported by the fact that *Xcc* isolates from different geographic regions can be genetically homogenous (Lange et al., 2015). On a more local scale, *Xcc* can be transmitted by wounding and water dispersal, a major source being the overhead watering of vegetable brassica seedlings in the glasshouse before transplant to the field. The bacteria can also persist between brassica crop rotations in the soil and crop debris for up to two years, depending on climatic conditions (Silva Júnior et al., 2020; Gazdik et al., 2021) and in weed hosts like Sheperd’s purse and fanweed. However, studies have shown that weed isolates are genetically distinct from those isolated from crops (Ignatov et al., 2007) and most are unable to cause disease in vegetable brassicas (Krauthausen et al., 2018). This suggests that weeds are potentially not a major inoculum source. It has been suggested that *Xcc* can be transmitted by insects, but this has yet to be proven (Vicente and Holub, 2013; An et al., 2020).

### 2.3. Diagnostics

As seeds are the primary *Xcc* inoculum source they are usually tested to prevent transmission. Seed-extracts are cultured on medias such as Fieldhouse-Sasser, mCS20ABN and King’s B (Koenraadt et al., 2005) where *Xcc* forms yellow-mucoid colonies (Figure 3D). There cultures are then confirmed by serology (Alvarez, 1994), PCR (Berg et al., 2005) or fatty acid methyl ester analysis (FAME) (Massomo et al., 2003). Positive results are then followed up with host plant assays to test pathogenicity (International Seed Federation, 2019). As black root causes distinct symptoms, often diagnosis is based on visual symptoms alone. However, at the point of symptom development, infection is already well established and consequently the effectiveness of control methods is limited. Currently, diagnostic methods are destructive, lab-based and time-consuming so there is a need for the development of rapid in-field diagnostics, e.g., loop-mediated isothermal amplification (LAMP) and Lateral Flow Tests (LFTs), which have been developed for *Xanthomonas* pathogens of other crops (Hodgetts et al., 2015; López-Soriano et al., 2017; de Paiva et al., 2019).

Some generalized tools for bacterial disease identification and management have been produced for brassicas in Europe, e.g., CropMonitor Pro, Taranis and xavario™ SCOUTING (SmartProtect, 2023). However, there are no specific forecasting tools for black rot incidence and disease severity. Development of such a tool that incorporates environmental conditions (temperature, humidity, rainfall, etc.), *Xcc* race-type, crop cultivar., watering systems and geographical region could be beneficial in preventing and controlling *Xcc*.

### 2.4. Prevention and management

#### 2.4.1. Farm practices

It is routine for brassica seed to be tested and where possible, only certified *Xcc*-free seed should be sown. Where farm saved seed is used it should be sent for testing and at a minimum sterilized prior to sowing. It is commonly recommended to sterilize brassica seed in hot water (50°C) for 20-30 min and this has been shown to reduce disease levels in the field (Roberts et al., 2022). However, hot water treatment alone does not completely eradicate *Xcc* on the seed and hydrogen peroxide washes are more effective and do not impact germination (Sanna et al., 2022). Similarly, UV-C treatment of seeds and plants has been shown to reduce disease incidence, but optimal ranges were narrow 2.5–3.6kJm^−2^; lower doses were not effective in eliminating *Xcc* and higher doses were detrimental to germination and yield (Brown et al., 2001). The application of UV-C to field crops would also be challenging.

The AHDB (2023a) recommend that brassicas should be sown in sterilized or new pots and ideally watered using an ebb and flow system that reduces water splash transmission of *Xcc* caused by traditional over-head systems. Inter-cropping with *Xcc* non-hosts like lettuce and legumes also has the potential to reduce transmission via water splash in the field by reducing crop density. However, this method is not widely practised and has been explored in the context of nutrient acquisition and nitrogen fixation but not black rot disease suppression (Stavridou et al., 2012; Jeromela et al., 2017). The AHDB also recommend that brassicas should be grown in 3-year field rotations to avoid build-up of *Xcc* in the soil from infected crop debris, which should be removed and burned directly after harvest and not ploughed into the field. It is still recommended to remove cruciferous weeds from the field, even though they are no longer thought to be a major inoculum source (Ignatov et al., 2007; Krauthausen et al., 2018). Field equipment, e.g., tractors and sprayers should be sterilized and used in uninfected areas first to prevent transmission through wounding. Finally diseased material should not be stored to prevent contamination and potential loss of the whole consignment.

#### 2.4.2. Chemical control

There are many chemical control options for *Xcc*, but they are generally ineffective as they are applied when symptoms are visible, and thus disease already established. Moreover, they are also damaging to the environment and human health. A study by Krauthausen et al. (2011) showed that benzoic acid and copper hydroxide sprays were only 12 and 32% effective, respectively, in reducing transmission in cauliflower transplants watered by overhead systems. Other studies on copper containing chemicals and pesticides (CuSO_4_, Kocide and copper nanoparticles) have shown a range in effectiveness from no control (Lenka and Ram, 1997) to reduced disease severity (Dong et al., 2020) and enhancing other treatments when used in combination (Patikarnmonthon et al., 2010; Nuñez et al., 2018). The most effective chemical treatment in the Krauthausen et al. (2011) study was enrichment of the overhead irrigation water with chlorine dioxide which reduced transmission by 94%. Antibiotics like streptocycline, streptomycin, oxytetracycline, chloramphenicol and rifampicin used as seed treatments and sprays have been shown to be more effective at controlling *Xcc* (Lenka and Ram, 1997; Bhat and Masoodi, 2000). However, the use of antibiotics and copper compounds to control bacterial plant pathogens can co-select for antimicrobial resistances (AMR) and so poses a serious threat to human health. Despite this, many countries do not regulate the use of antibiotics in crops, although many EU countries and the UK do not have any licensed as plant protection products (Haynes et al., 2020).

#### 2.4.3. Biological control

There are very few biocontrol agents for black rot approved for commercial use. One such product is Actigard, a broad acting pesticide that induces systemic acquired resistance (SAR) and has been shown to reduce black rot incidence by 51% (Langston and Cummings, 2003). Fungal biocontrol agents like *Trichoderma* spp. work in a similar way by inducing SAR and have been shown to control *Xanthomonas* spot of tomato (Fontenelle et al., 2011). Whether this phenotype translates to brassica crops needs further investigation. The *Trichoderma* metabolite 6-pentyle-α-pyrone (6PP) and botanical extracts also look promising as controls as they have been shown to inhibit *Xcc* biofilm formation and bacterial growth (Sain et al., 2007; Papaianni et al., 2020).

For more precise targeting of *Xcc*, bacteriocins and phages can be used. Bacteriocins are small proteins produced by bacteria to kill other closely related bacteria, helping them establish a niche in their environment (Daw and Falkiner, 1996). Bacteriocins targeting other Xanthomonads have been identified (Pham et al., 2004; Bonini et al., 2007; Marutani-Hert et al., 2020) but none that target *Xcc*. These could be predicted through sequencing and analyses of *Xcc* and other closely related Xanthomonads and could be applied as seed or spray treatments. Conversely, numerous phages have been identified that target *Xcc* (Orynbayev et al., 2019; Holtappels et al., 2022), but generally they are fragile and easily degraded by environmental UV-B. A study by Orynbayev et al. (2020) showed that skimmed-milk gave phages the best protection against UV-B. A subsequent study by Nuñez et al. (2018) showed that milk-based products alone can reduce *Xcc* severity in kale under field conditions by up to 44%, as well as simultaneously improve nutritional value. So, a combination of milk-based products and phage could cumulatively improve *Xcc* control. While promising, the majority of biocontrols for black rot are in the research and development phases and require considerably more work to realize a marketable product.

#### 2.4.4. Resistant cultivars

Host resistance provides an alternative to chemical control but is challenging due to the genetic diversity and race structure of *Xcc*. Several brassica varieties are reported to be resistant or have field tolerance to black rot (Table 1) but their effectiveness against different *Xcc* races is not known and is limited in certain crop types like savoy cabbage and cauliflower. Over 30 resistance sources have been reported in the literature and several QTLs have been mapped (Taylor et al., 2002; Singh et al., 2018; Shaw et al., 2021). Most of the resistances identified are race-specific, mostly to the prevalent races 1 and 4, and in most instances are from other *Brassica* spp. (*B. rapa*, *B. nigra, B. carinata*, *B. napus* and *B. juncea*) so require introgression into commercial vegetable brassica (*B. oleracea*) types, where currently there is limited resistance. This will be time consuming as it can take >10 years to introgress traits using traditional breeding methods. This could be accelerated with gene-editing/modification techniques, but this would require further mapping to refine QTLs and identify specific resistance genes to target. Race-specific diagnostics of *Xcc* will help inform the deployment of the correct resistances and resistance stacking may be necessary in areas where multiple races are prevalent.

**Table 1:** Black rot (BR), clubroot (CR), downy mildew (DM), and turnip yellows virus (TuYV) resistant *Brassica* varieties.

Species	Crop type	Variety	Resistant to:			
			BR	CR	DM	TuYV
*Brassica oleracea* var. *gemmifera*	Brussel sprouts	Attis	X			
		Doric F1	X			
		Nautic F1				
		Crispus F1		X		
		Cronus F1		X		
		Might hybrid			X	
*Brassica oleracea* var. *capitata*	White cabbage	Bravo F1	X			
		Kilaton F1		X		
		Kilaxy F1		X		
		Kilazol		X		
	Red cabbage	Red Dynasty F1	X			
		Scarlet	X			
		Lodero		X		
	Savoy cabbage	Tourmaline F1	X			
		Alcosa F1			X	
		Famosa F1			X	
*Brassica rapa* var. *pekinensis*	Napa or Chinese cabbage	Blues F1	X		X	X*
		Barrel Head Hybrid		X	X	
		Bilko F1		X		
		China Gold		X		
		Emiko F1		X		
		Pacifiko		X		
		Spring Crisp		X		
		Wawa Tsai		X		
		Yuki		X		
		China Express			X	
		Haku			X	
		Norang Bom			X	
*Brassica oleracea* var. *italica*	Broccoli	Bonarda F1	X			
		Burney F1	X		X	
		Gemini F1	X			
		Greenpak 28	X			
		Marathon F1	X		X	
		Millennium F1	X			
		Tendergreen F1	X			
		Emerald Jewel F1		X	X	
		Monclano F1		X	X	
		Athlete F1			X	
		Aquiles F1			X	
		Avenger F1			X	
		Babilon F1			X	
		Belstar F1			X	
		Diplomat F1			X	
		Emerald Crown			X	
		Emerald Pride			X	
		Everest F1			X	
		ExpGreen Gold F1			X	
		Green Magic F1			X	
		Gypsy F1			X	
		Hurdle F1			X	
		Imperial F1			X	
		Maximo F1			X	
		Patriot F1			X	
		Seabiscuit F1			X	
		Te You Flowerly			X	
		Triathlon F1			X	
*Brassica oleracea* var. *botrytis*	Cauliflower	Clapton F1		X		
		Clarify F1		X		
*Brassica napus*	Oilseed rape	Caletta				X
		Amalie				X
		Ambassador				X
		Annalise				X
		Architect				X
		Artemis				X
		Aspire				X
		Aurelia				X
		Darling				X
		Dazzler				X
		Ludger				X
		Temptation				X
		Allessandro				X
		Feliciana				X
		Atora				X
		Dominator				X
		Cadran				X
		Coogan				X
		Addition				X
		DMH440				X

Modified from CornellCALS (2022). *Virus resistance stated but not specified.

## 3. Clubroot

### 3.1. Host and impact

Clubroot disease is an age-old disease that was believed to affect cultivated brassicas in Europe at least as far back as the 13th century and quite possibly as early as the Roman era. It has been ascribed to many causes but in 1873, the protist pathogen *Plasmodiophora brassicae* was identified as the causal agent (Chupp, 1934). The disease is especially prevalent in, but it is not limited to, moist and temperate areas. The epidemics are developing rapidly as the dietary, and industrial significance and cultivation of *Brassica* crops increases. The exact origin of clubroot disease is unknown, however, it is believed that the pathogen traveled from Europe to other parts of the world by means of infected plants used as animal fodder (Gibbs, 1931). Clubroot disease is a global problem, it is reported to be distributed in more than 88 countries and can result in a 10–100% reduction in yield (Saharan et al., 2021). In some countries, this disease is widespread and significantly affects yield losses (Table 2).

**Table 2:** Worldwide food loss caused by clubroot disease of brassicas.

Country	Yield loss (%)	References
Australia	30–60	Donald and Porter (2014)
Canada	30–100	Hwang et al. (2011)
China	14–80	Chai et al. (2014), Ren et al. (2016), and Feng et al. (2013)
Czech Republic	10–100	Řičařová et al. (2016)
Finland	5–16	Rastas et al. (2012)
India	30–70	Bhattacharya et al. (2014)
Nepal	35–40	Timila et al. (2008)
Poland	25	Korbas et al. (2014)
Sweden	82	Wallenhammar et al. (2014)
UK	50	McGrann et al. (2016)

**Figure 4:**
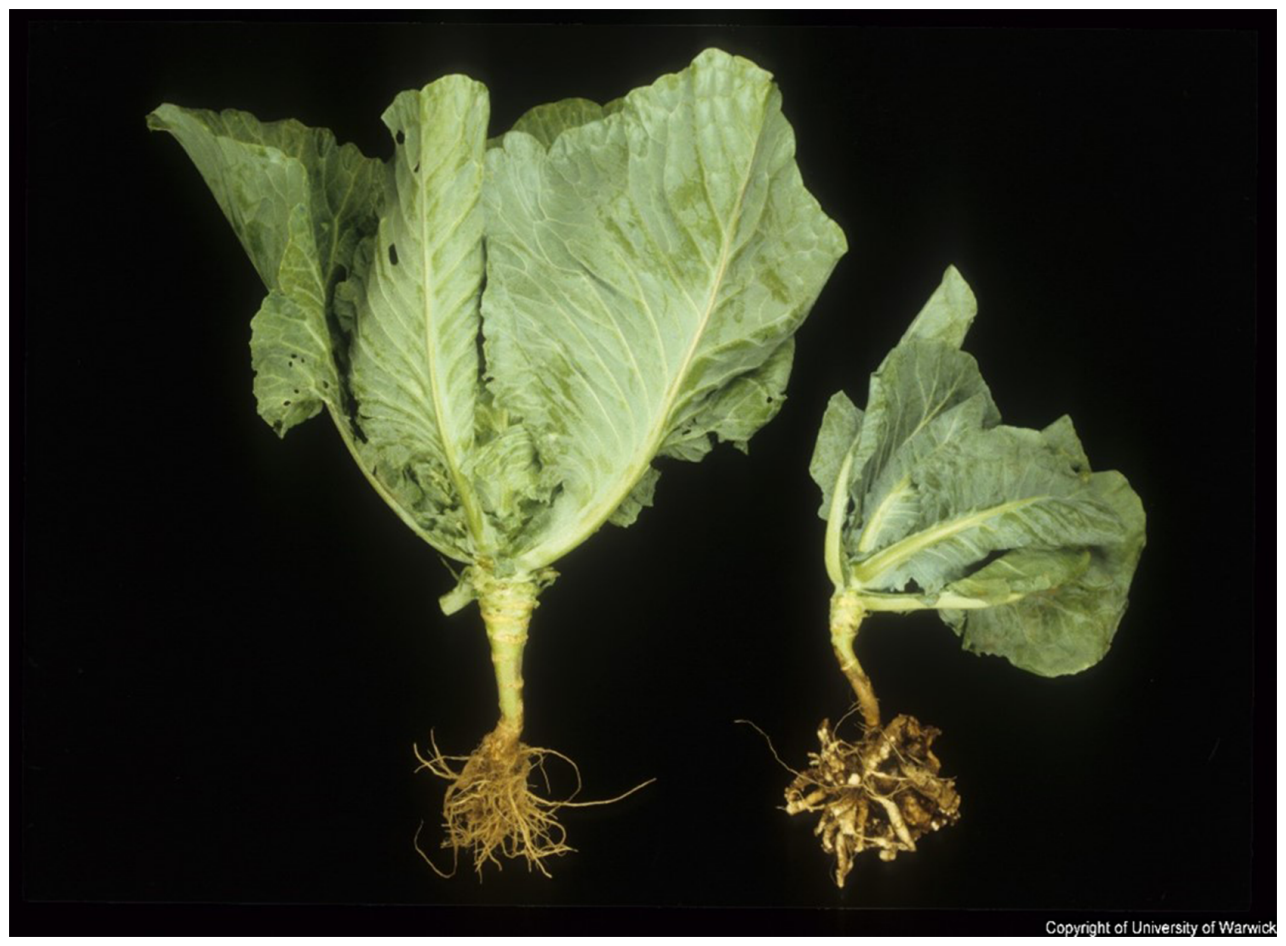
Symptoms of clubroot infection in *Brassica* caused by *Plasmodiophora brassicae* (photograph by John Walsh). The left plant is healthy, and the right plant is infected with *P. brassicae* and shows classic root gall but no foliar symptoms.

A classical symptom of clubroot infection is the formation of white-colored solid root galls which later discolor and rot (Figure 4). Above-ground symptoms appear only in the final stages of infection and include stunting and yellowing of the leaves. Under dry conditions, severe galling can inhibit water uptake from the soil and cause wilting. In severely infected areas the whole crop can fail (Saharan et al., 2021).

It is believed that *P. brassicae* is a potential pathogen for all 330 genera and 3,700 species in the *Brassicaceae* family. Apart from most vegetables, oil, and fodder crops, *P. brassicae* also infects cruciferous weeds such as Shepherd’s purse, stinkweed, wild mustard and ornamental plants such as *Matthiola* spp. and *Cheirantus cheiri* (Buczacki, 1979; Howard et al., 2010; Saharan et al., 2021). The wide host range of *P. brassicae* make it particularly difficult to manage. The incidence and virulence of the pathogen is greatly dependent on the host and is considered a ‘Disease of cultivation’.

### 3.2. Pathogen lifestyle and transmission

As a soilborne pathogen, *P. brassicae* presents abundant challenges to agriculturists and biological scientists due to its complex lifecycle (Saharan et al., 2021). The pathogen is obligate, intracellular, non-axenic, microscopic, single-celled, and soilborne with dormant, well-protected resting spores. These spores are composed of layers of chitin and carbohydrates (Moxham and Buczacki, 1983) and can survive for up to 20 years in the soil (Dixon, 2009). Consequently, clubroot disease is almost impossible to eradicate once it is established.

*Plasmodiophora brassicae* infection can be broadly divided into primary and secondary infections. During primary infection resting spores germinate in the presence of brassica root exudates in the soil (Bochow, 1963, 1965). However, germination can be triggered by root exudates from other plants such as poppy (*Papaver* spp.), strawberry (*Fragaria* spp.), and ryegrass (*Lolium* spp.) (Craig, 1989). The resting spores germinate into a primary zoospore which has two flagella, and it enters the host via root hairs (Kageyama and Asano, 2009). This is the only stage of *P. brassicae* that is considered unprotected (without spore walls) (Burnett et al., 2019). The conducive environment for the primary zoospores to survive and enter the hosts are acidic pH (<6.8), low calcium, nitrogen in the form of ammonia and water logged soil with a temperature of greater than 15°C (AHDB, 2020). The primary zoospores colonize the root hair and produce primary plasmodia inside the host. Secondary zoospores are then released into the soil.

Secondary zoospores are the starting point for secondary infection, they form secondary plasmodium in host cells and neighboring cells undergo extreme hyperplasia (cell division) and hypertrophy (cell expansion), resulting in the formation of galls. The gall formation affects the vascular tissues in the root which limits the transport of nutrients and water to aerial parts of the infected plants causing yellowing and stunting (Ludwig-Müller et al., 2009). These symptoms only appear at the later stage of infection. Disintegration of the galls releases resting spores into the soil to start the infection cycle again.

### 3.3. Diagnostics

The greatest challenge of managing clubroot disease in the field is the lack of rapid and reliable detection methods and/or tools. Historically, clubroot infection was detected by the “plant bioassay method,” where bait plants are grown in suspect soil for 5–6weeks and subsequently examined for root galls (Dixon, 2009). Although this method is reliable and inexpensive, it is extremely time-consuming, labor intensive, and the results are highly influenced by environmental factors (Dixon, 2009). Alternative diagnostic methods include microscopic observation of host tissues, fluorochrome staining of *P. brassicae*’s resting spores in the soil (Takahashi and Yamaguchi, 1988, 1989) and the application of monoclonal antibodies in serological methods (Dixon, 2009). PCR can also be used to check seeds and tubers for *P. brassicae* (Rennie et al., 2011). These methods are reliable and sensitive, however, they are lab-based, require expensive equipment and technically skilled persons.

Global Positioning System (GPS), and Geographic Information System (GIS) have been used to help visualize and track the spread of *P. brassicae* around infected regions (Saharan et al., 2021). CLIMEX is a forecasting tool for clubroot distribution and disease severity and was developed in Canada (Howard et al., 2010). It uses temperature and moisture content data but requires further refinement to include various other soil parameters, e.g., nutrient content presences of alternative host species, *P. brassicae* pathotypes, crop cultivar and geographical region to be an effective tool to control clubroot disease. A major hinderance in developing forecast tools is the lack of previous data, farmers are often aware of the factors that influence clubroot disease but most of them fail to record these (Saharan et al., 2021). Further development of geographic specific modeling tools is required to effectively control clubroot disease in brassicas.

### 3.4. Prevention and management

#### 3.4.1. Farm practices

Clubroot disease can be controlled by clean farm practice. Any activity that moves the soil or plants from an infected field to another has the potential to spread *P. brassicae*. It is important to disinfect machinery, equipment, vehicles, tools, footwear, seeds, plant materials, water and/or soil to prevent the spread of clubroot. Seed contamination can cause long-distance dissemination of *P. brassicae* and so seed cleaning is particularly important. Hwang et al. (2008) showed that most commercial disinfectants except sodium hypochlorite solution (1–2%) were ineffective at deactivating *P. brassicae* spores after a 20–30 min contact time. The study showed that sanitization can limit the *P. brassicae* pathogen but not eradicate it. Timing of pre-and post-harvest weed management is also crucial for controlling clubroot disease because the cruciferous weeds could act as a potential host for *P. brassicae* (Friberg et al., 2005; Ahmed et al., 2011).

Soil solarization is one of the methods used in tropical regions to control clubroot disease (Jacobsohn et al., 1980). In this technique, the infected soil is covered by a polyethene sheet to trap the heat and inactive *P. brassicae* spores. This technique is efficient in sandy soil with low disease pressure but requires the addition of fumigation agents in clay soils and when the disease pressure is high (Donald and Porter, 2014). Acidic soil often favors clubroot development and hence soil liming is considered an effective practice to control the disease. In this method, the soil pH is maintained around 7.2 or higher by the addition of lime (Myers and Campbell, 1985; Rastas et al., 2012). The lime is available in slow-acting and fast-acting forms. The slow-acting forms of lime such as agricultural lime and dolomitic lime are applied during autumn at 5–7.5 t/ha to allow time for them to react and diffuse so that soil is prepared for spring planting (Hwang et al., 2008). Fast-acting lime such as hydrated lime and quick lime is applied before planting. An important aspect to consider in this method is that liming effect is greatly varied depending upon the soil type. This method is inapplicable in soil with high buffering capacities and in ‘lime non-responsive’ soil (Myers et al., 1981).

High soil moisture content at 25°C favors clubroot (Feng et al., 2010; Sharma et al., 2011) and so it is recommended where possible to avoid these condition at the early stage of seedling development, whereas older plants are less affected. Gossen et al. (2012) showed that early sowing of *Brassica* plants reduced the disease severity by 10–50% and increased the yield by 30–58%. Longer crop rotations of more than 3 years are recommended to reduce disease build-up in the field. Further intercropping with Yellow Sarson and flaxseed has also been shown to reduce clubroot severity by 30–50% (Poulton, 1990). The presence of hydrogen cyanide in root exudates is additionally hypothesized to inhibit the clubroot pathogen (Poulton, 1990). Various farm practices are recommended for clubroot disease however it is greatly dependent on the local environment.

#### 3.4.2. Chemical control

Very few chemicals are known to exhibit consistent control of clubroot disease in *Brassica*. Among them, the most efficient and widely used chemical is mercurous chloride (Calomel™). However, it has been withdrawn from the market due to its mercury toxicity and persistence in the environment for long periods (Saharan et al., 2021). Currently, the most used chemicals to control clubroot disease are benzimidazoles and their precursors. Seed treatments have been shown to reduce the severity of the disease and increase yield (Hwang et al., 2011) but another study has shown that they only reduce the dissemination of *P. brassicae* via seed and give no protection in the field (Rod, 1992). The major limitation in chemical control is the product delivery to the infected parts and secondly, most of the chemicals are environmental pollutants. An effective way to reduce the *P. brassicae* inoculum in the field is to fumigate the soil with dithiocarbamates such as metham sodium and dazomet. When these products get into contact with moist soil, they are converted to volatile compounds that diffuse through the soil and act as a fungicide. The fumigation activity is greatly dependent on the moisture content (the optimal moisture content is around 10–30%) and the soil type (Donald and Porter, 2014). The downside of utilizing fumigants is that it causes localized air pollution.

The application of calcium cyanamide fertilizer plays an important role in IPM. The slow-release nature of the fertilizer reduces nitrate pollution and makes calcium readily available, which in turn encourages microbial diversity and suppresses soil-borne pathogens like *P. brassicae* (Dixon, 2009). Various researchers have identified calcium cyanamide as an effective control for soil-borne pathogens (Tremblay et al., 2005; Donald and Porter, 2014).

#### 3.4.3. Biological control

The weakest link in the lifecycle of *P. brassicae* is the zoospore stage and so, many disease management strategies target this stage. Using bait crops in combination with liming could control clubroot disease (Harling and Kennedy, 1991). Bait crops, like radish stimulate the germination of resting spores to zoospores (Bhattacharya et al., 2014). However, this strategy is only advised when the disease severity is at a lower-moderate level. The greatest disadvantage of this method is that it is time-consuming, most bait crops are hoed in after 6weeks and during this time the land is out of production. The application of seaweed (*Posidonia australis*) has also been shown to stimulate the germination of *P. brassicae* resting spores and reduce the disease severity (Tilston et al., 2002). There are no commercial stimulants on the market that could be used for inducing resting spore germination.

Many common microorganisms residing in the rhizosphere and root cortex of common vegetables and field crops do not flourish in the presence of *Brassica* species (Usuki and Narisawa, 2007). This unique characteristic of *Brassica* crops limits the application of common biocontrol agents that are rhizosphere colonizers and producers of antimicrobial compounds (Saharan et al., 2021). The most common biocontrol agents for clubroot disease are *Bacillus subtilis* (Serenade^®^), *Gliocladium catenulatum* (syn. *Clonostachys rosea* f. *catenulate*) (Prestop^®^), *Streptomyces griseoviridis* (Mycostop^®^), *S. lydicus* (Actinovate^®^), and *Trichoderma harzianum* (RootShield^®^) (Sharma et al., 2011; Saharan et al., 2021). The formulation plays a crucial role in the survival and delivery of biocontrol agents. *B. subtilis* has an advantage over other biocontrol agents in respects to formulation because it is capable of producing spores under adverse conditions (Schisler et al., 2004). Serenade^®^ and Prestop^®^ are more effective under lower *P. brassicae* inoculum pressure, with 85–100% reduction in disease severity (Peng et al., 2011). However, Mycostop^®^ was more effective than Serenade^®^ and Prestop^®^ at the higher disease pressure (Adhikari, 2010).

Plant bio-stimulants like algae extract, amino acids, and phosphonate are also used to suppress clubroot disease. Kammerich et al. (2014) showed that two commercial bio-stimulants Frutogard^®^ (liquid) and PlasmaSoil^®^ (granules) reduced disease severity and gall size on *B. rapa* (Chinese cabbage) and *B. napus* (oilseed rape). However, few studies have been conducted at the field level to test effectiveness of biocontrol agents against clubroot disease and most of the studies presented in this section were conducted in a controlled environment.

#### 3.4.4. Resistant cultivars

Sources of resistance to *P. brassicae* have been identified in various parts of the world. Breeding is considered the most effective disease management strategy for clubroot. Resistance to *P. brassicae* can vary from broad-spectrum resistance, effective against several races and pathotypes to highly specific to one strain. Numerous resistance varieties have been developed around the world (Table 1) but their effectiveness against different *P. brassicae* races and pathotypes is not known and is limited in crop types like broccoli and savoy cabbage.

Genetic mapping has revealed several dominant loci, twelve in *B. rapa*, >22 QTLs in *B. oleracea* and > 19 QTLs in *B. napus* for clubroot resistance to different pathotypes of *P. brassicae* (Saharan et al., 2021). The first resistance cultivars were Mendel and Tosca. These cultivars are heterozygous hybrids with pathotype-specific resistance. Unfortunately, the Mendel host resistance was rapidly broken down in Germany, France and the UK as a result of *P. brassicae* quickly adapting to overcome the resistance (Saharan et al., 2021). This is an example of why resistance cultivars must be used with care; they should be used in combination with other resistances and management practises to minimize the risk of resistance breakdown. The Mendel and Tosca resistances have been shown to be highly variable, sometimes being in-effective in some regions and to some pathotypes of *P. brassicae* (Peng et al., 2013, 2014).

It is expected that the market will receive more resistance varieties in the next decade due to the development of molecular techniques (Peng et al., 2014; Saharan et al., 2021). However, these resistances may break down without sustainable agronomic practices and pathogen resistance management strategies.

## 4. Downy mildew

### 4.1. Host and impact

The term downy mildew was first used in the USA to describe fungus that appeared as white-colored outgrowths caused by the proliferation and fructification of mycelium on the surface of green and necrotic plant tissues (Saharan et al., 2017). Downy mildew is caused by *Hyaloperonospora parasitica* syn. *Paronospora parasitica*, an obligate biotrophic oomycete in the family *Peronosporaceae,* a distinct group of pathogens classified as *Mastigomycotina* in the order *Peronosporales* (Voglmayr, 2003; Göker et al., 2009). *H. parasitica* remains understudied because it is difficult to culture in artificial media and collect pathogen samples due to its obligate nature. It originated from northern parts of America, and quickly adapted to European weather. It is currently widespread around the world, with reports from 93 countries across 6 continents (CABI, 2022b).

**Figure 5:**
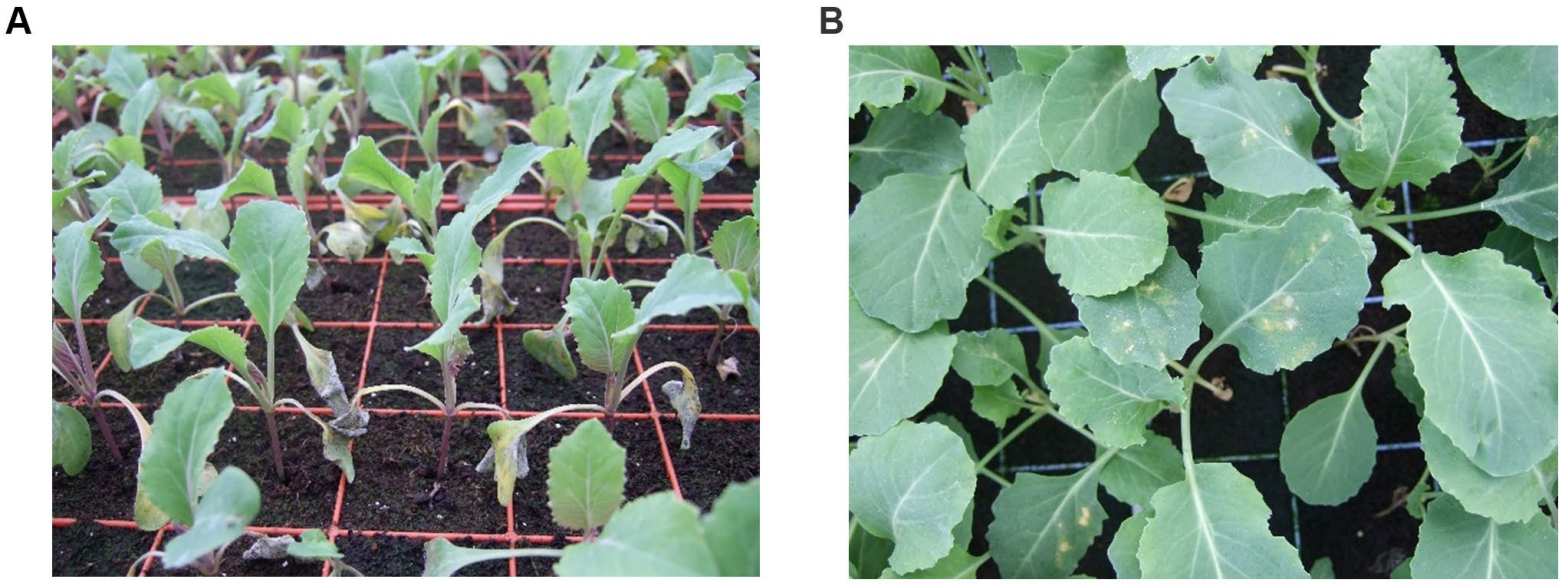
Symptoms of downy mildew infection caused by *Hyaloperonospora parasiti*ca on *Brassica oleracea* (photographs by Joana G. Vicente). **(A)** White powdery and wilted cotyledons of infected plants. **(B)** Yellow and white powdery lesions on the top side of older infected leaves associated with the veins.

The host range for downy mildew is vast, particularly in the *Brassicaceae* where it infects 50 genera and more than 100 species (Gaumann, 1923). *Hyaloperonospora parasitica* infects more than 20 economically important *Brassica* crops, with the greatest impact on mustard (*B. nigra* and *B. juncea*) and oil seed rape (*B. napus*) (Saharan et al., 2017). The pathogens broad host range make it difficult to control. The symptoms of downy mildew infection of brassica are usually seen first in the cotyledons, followed by the true leaves with the appearance of pale green-yellowish spots, which are angular in shape and bound to the leaf vein (Figure 5). Often these symptoms are misinterpreted as nutrient deficiencies. The yield losses due to downy mildew disease alone are difficult to quantify because most of the time it is accompanied by white rust disease and viral diseases (Saharan et al., 2017), the yield loss due to the combined infection were reported to be around 23–55% (Saharan et al., 2017). Meena et al. (2016) reported yield losses of 11% (143.2 kg/ha) in Indian mustard plants in 2014 due to downy mildew. Disease severity also varies with the host plant.

### 4.2. Pathogen lifestyle and transmission

*H. parastica* is an obligate parasite of brassicas and has primary (sexual) and secondary (asexual) infection stages. Primary infection starts with the germination of sexually produced soilborne oospores in the presence of host plants and under conducive climatic conditions (Chang et al., 1963; Shiraushi et al., 1975). The oospores form germ tubes that penetrate the plant roots. Mycelium then form, growing intracellularly to produce haustoria that penetrate the cells for nutrient acquisition (Slusarenko and Schlaich, 2003). After 1–2 weeks, conidiophores grow out of stomata and release asexual conidiospores that are dispersed by wind and water splash to cause secondary infection of nearby hosts (Lucas et al., 1995). Conidiospores also produce germ tubes which penetrate the lower surface of the leaves (Chou, 1970; Achar, 1995) but sometimes infection can occur through the stomata. Concurrently, sexual oospores are produced in high number in the leaves, which then drop and infest the soil for further primary infection. As well as in the leaf, oospores have been found on the seed surface and in the pericarp and embryo. However, seed transmission has been shown to be extremely low 0.4–0.9% in rapeseed and mustard (Saharan et al., 2017) and so it is not considered an important route of transmission.

Generally, downy mildew on *Brassica* crops is more problematic when the temperature is between 10–15°C with high moisture conditions. However, the empirical data for disease development in the field is still lacking. Studies conducted in a controlled environment show that different developmental stages of the pathogen require different optima, conidial germination (8-12°C), cell penetration (16°C) and haustoria formation (20-24°C) (Chu, 1935; Felton and Walker, 1946). Oospores can survive for long periods (up to 5 years), without a host or during unfavorable conditions. Conversely, the survival as mycelium and conidial forms are greatly dependent on the environmental conditions (Saharan et al., 2017). Lin and Liang (1974) reported that conidia in the infected leaves of kohlrabi leaves survived for 10 days under warm field conditions and that the conidia could survive up to 110 days in a conducive environment. Marked reductions in the survival of conidia were observed in moist and dry soil in warm conditions (up to 22 days) (Krober, 1970).

### 4.3. Diagnostics

To date, diagnosing downy mildew mainly relies on observation and recognition of symptoms either by eye or using lenses (Slusarenko and Schlaich, 2003). The common serological methods for detection are ELISA and lateral flow assays. However, the major limitation of these methods is the cross-reactivity of antibodies with other oomycete species (Salcedo et al., 2021). While isoenzyme methodology can be used for diagnostics, this requires a large amount of pathogen material (Bonde et al., 1984). Moreover, these methods have been developed in other plant species and limited studies have been conducted in brassicas. The most reliable technique to detect *H. parasitica* is using molecular technologies. These methods detect the presence of the pathogen with a specific DNA marker, and includes approaches such as restriction fragment length polymorphism (RFLP), amplified fragment length polymorphism (AFLP), random amplified polymorphic DNA (RAPD), micro- and minisatellites (Schroeder et al., 2013; Wallace and Quesada-Ocampo, 2017). Although accurate, these are lab-based techniques, are relatively expensive requiring specific equipment and skilled personnel to interpret the results.

Recently, researchers have developed portable sequencers to enable in field detection of *H. parasitica* but they require further investigation to determine the sequencer’s sensitivity and reliability (DeShields et al., 2018; Salcedo et al., 2021). Additionally, high-resolution thermal and multi-spectral imagery has been used to detect downy mildew (*Peronospora arborescens*) in poppy fields (Landa et al., 2007). This technology could be translated to downy mildew detection in brassicas. However, these new diagnostic methods are only at the R&D level.

A huge knowledge gap exists in the epidemiology and forecasting of downy mildew. This is largely due to the complex life cycle of *H. parasitica* and that downy mildew is almost always present with other disease like white rust. Various forecasting models have been developed around the world but have not been robustly tested. For instance, a model developed in India gave a 21 and 36% deviation from predicted and actual downy mildew disease incidence in 1991–1992 and 1992–1993, respectively (Mehta and Saharan, 1999). The same model predicted a deviation of 66 and 16% in the same years for white rust. These large deviations for both diseases show that the model needs further improvement for best fit. Often a prediction model developed for one location might not fit another location. Hence, the model must be tested in multiple locations and for longer periods as well as consider microclimate, *H. parasitica* pathotype and crop cultivar to develop an effective tool (Mehta, 2014).

### 4.4. Prevention and management

#### 4.4.1. Farm practices

One of the greatest challenges in controlling downy mildew is that it has a very short latent period. Raising disease-free seedlings is considered one of the crucial steps in downy mildew prevention and management. Good hygiene practices include sterilizing the glasshouse between each crop and raising plants in plastic or concrete platforms to avoid contact with the soil. To prevent secondary infection, it is recommended to remove cruciferous weeds from the field as they can act as an inoculum source. Infected plants and debris should also be removed quickly and destroyed to prevent contaminating the soil with long-lasting oospores which can lead to primary infections (RHS, 2023). In addition, it is recommended to avoid overhead irrigation systems, maintain well-drained soil, have long crop rotations (minimally two years), reduce planting density to reduce the relative humidity and water in the early morning to enable adequate time to dry to reduce the dispersal of spores (Sherf and MacNab, 1986). Koul and Singh (1999) showed that the removal of 50% of lower leaves within 60 days of the crop age reduced disease severity as it helped to reduce the relative humidity around the plants. They also showed that early sowing reduced disease severity in *B. juncea* grown in India because the plants matured before the pathogen could reach epidemic form.

#### 4.4.2. Chemical control

Although not a fungus, downy mildew disease is primarily controlled by fungicide treatments like metalaxyl. Resistance to metalaxyl was reported in *H. parasitica* within 40 years of its introduction (Crute et al., 1985; Crute and Pink, 1996). Despite this, metalaxyl is still used for the control of downy mildew. Seed treatment with Apron (mixture of metalaxyl and captan) at the rate of 2 g/Kg has been shown to give 96% disease control in cauliflower and generally increased yield when used in combination with 1–2 foliar sprays (Verma and Thakur, 1989). White et al. (1984) showed that cauliflowers seed-treated with Apron did not develop disease symptoms, even after the artificial inoculation. However, metalaxyl is persistent and was present in cabbage leaves up to 4 weeks after sowing from treated seeds (Crute et al., 1985). An effective and economical schedule for the application of seed treatment and/or foliar sprays must be tailored for each local condition and crop. For instance, with mustard plants grown in India it is recommended to treat seed with Apron followed by three foliar sprays at 30 days intervals to control downy mildew (Koul and Singh, 1999). Since sporulation of the pathogen occurs mainly at the night and is disseminated during the morning, it is recommended to apply fungicides at the end of the day (AHDB, 2020).

Apart from seed treatment and foliar sprays, fungicides are also applied to the soil before sowing. The application of fungicides such as prothiocarb and fosetyl-aluminium as a soil drench with 50% seed emergence has controlled the disease progression in cauliflower and radish plants (Saharan et al., 2017). The application of granular metalaxyl pre-sowing also exhibited promising results on broccoli (0.56 a.i/ha) and cauliflower (0.28 a.i/ha) (Chiu, 1959). Compost treatment is also effective in controlling downy mildew disease, in the UK, module-raised cauliflower plants are grown in compost containing metalaxyl, milfuran with manganese zinc dithiocarbamate or propamocarb to control downy mildew. Although fungicides are effective at controlling downy mildew, they have their disadvantages (1) they persist in plants and will have associated toxicity (2) emergence of pathogen resistance (3) costly production and application processes as well as potential unknown environmental impacts.

Biochemicals such as salicylic acid and its derivatives can activate systemic acquired resistance in the host (Wang et al., 2007; Collier et al., 2011). Promising results were observed when S-methyl benzo [1,2,3] thiadiazole-7-carbothioate (a derivative of salicylic acid) was used to treat seeds of *B. oleracea* against *H. parasitica* (Molina et al., 1998). These biochemicals have a different mode of action compared to the conventional chemical control agents and when used as part of an integrated pest management strategy, would result in more durable control of downy mildew disease in *Brassica* crops (Jensen et al., 1998). However, further evaluation is required to determine the longevity and effectiveness of biochemicals for field use.

#### 4.4.3. Biological control

*Trichoderma harzianum* and *Pseudomonas* spp. are the most common biocontrol agents for downy mildew in *Brassica* crops. Both have been shown to have a similar ability to reduce downy mildew disease severity in *B. juncea* when used as seed treatments and foliar sprays, singularly and in combination (Meena et al., 2013). In the same study, it was shown that a seed treatment with aqueous garlic extract followed by foliar sprays significantly reduced downy mildew disease incidence and severity (Meena et al., 2013). Garlic juice and aqueous extract of garlic have also been reported to inhibit *H. parasitica* in radish plants (Bedlan, 1987). Additionally, avirulent stains of *H. parasitica* have been shown to induce downy mildew resistance in broccoli and cauliflower (Singh et al., 2002). Although promising, further research is required to see how applicable avirulent strains would be in a field setting. One potential risk is that they may exchange genetic material (e.g., through horizontal gene transfer) with pre-existing isolates in the field and as a result become virulent, thus exacerbating the problem rather than remedying it.

#### 4.4.4. Resistant cultivars

Compared to other diseases discussed in this review, downy mildew can sexually reproduce meaning that host resistance can be more easily overcome (Saharan et al., 2017). There are six pathotypes of *H. parasitica* which pose another challenge for resistance breeding (Coelho et al., 2012; Vicente et al., 2012). Fully and partially resistant varieties are available on the market or are under development (Ridout, 2018; Table 1), these are mainly broccoli types and there is limited or no resistance in Brussels sprouts, cauliflower and cabbage. Single gene resistance has been identified in various *B. oleracea* cvs. such as cauliflower, broccoli, kale and Brussels sprouts (Hoser-Krauze et al., 1984; Farnham et al., 2002; Carlsson et al., 2004) and also multi-gene resistances in in cauliflower, broccoli and cabbage (Jensen et al., 1999; Moss, 2002). However, they are race and geographic specific. Although downy mildew is considered one of the most devasting brassica diseases little research has been conducted to explore the development of resistance cultivars.

## 5. Turnip yellows virus

### 5.1. Host and impact

Turnip yellows virus (TuYV) syn. beet western yellows virus (BWYV) is a positive-sense, single-stranded RNA virus belonging to the genus *Polerovirus* in the family *Solemoviridae* (previously *Luteoviridae*, Scheets et al., 2020). BWYV was first reported in the USA in the 1950s as causing stunting and chlorosis of a broad range of plant species including sugar beet, brassicas and lettuce (Duffus, 1960). In the 1960s, BWYV-like isolates were reported in the UK that were serologically similar but not able to infect sugar beet (Russell and Duffus, 1970). These non-beet infecting isolates were reclassified as TuYV (Mayo, 2002). Subsequent molecular and sequencing analyses supports BWYV and TuYV as being different species (Hauser et al., 2000; Stevens et al., 2005).

**Figure 6:**
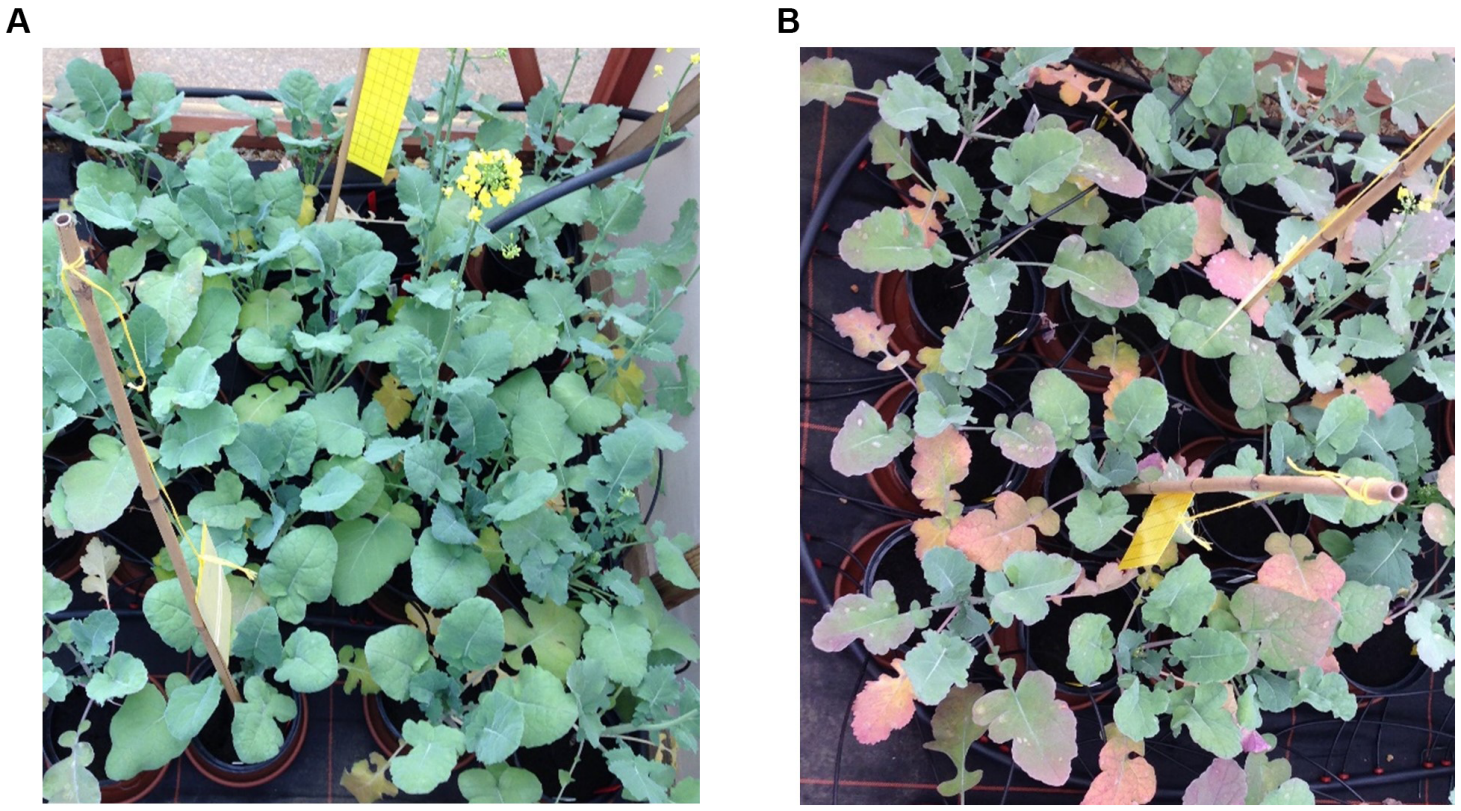
Symptoms of turnip yellows virus (TuYV) infection in oilseed rape (*Brassica napus*). **(A)** Healthy oilseed rape plants not infected with TuYV. **(B)** Oilseed rape plants infected with TuYV showing redding and purpling of the leaves.

TuYV has a broad host range infecting species from more than 16 plant families (Stevens et al., 2008). It is one of the main viruses infecting vegetable and oilseed brassicas and has been reported in 26 countries and 6 continents (Greer et al., 2021b; CABI, 2022a), but it is likely an underrepresentation and TuYV is probably present in other brassicas growing regions but not reported. TuYV infection can cause a range of non-specific symptoms in brassicas such as chlorosis, purpling and reddening of the leaf margins and stunting (Figure 6) but often infection can be symptomless. In oilseed brassicas, yield losses of 12–46% due to TuYV infection have been reported (Graichen and Schliephake, 1999; Jones et al., 2007; Nicholls and Stoddart, 2014) and in vegetable brassicas like Brussels sprouts, the virus can cause losses as high as 65% (Walsh et al., 2011). Furthermore, TuYV infection has been associated with tipburn, an internal disorder of stored white cabbage that can seriously impact post-harvest quality (Hunter et al., 2002).

### 5.2. Pathogen lifestyle and transmission

TuYV is transmitted by aphids in a persistent, circulative and non-propagative manner (Coleman, 2013). This means that once an aphid ingests TuYV from feeding on an infected plant, it carries and can transmit the virus for life, but the virus does not replicate within the aphid or pass to its progeny. Schliephake et al. (2000) showed that 17 species of aphid can transmit TuYV and that *Myzus persicae* (green peach-potato aphid) was the most efficient vector. In a study at Broom’s Barn Research Center, UK between 1994 and 2002, 72% of *M. persicae* caught in water traps tested positive for TuYV (Stevens et al. 2008). Furthermore, TuYV incidence in oilseed rape crops has been shown to be closely linked with the number of migrating *M. persicae* (Asare-Bediako et al., 2020). Thus, years with increased aphid numbers saw higher TuYV incidence, sometimes as high as 100%. There is also some evidence to suggest that TuYV infected plants release chemical volatiles that attract *M. persciae* to promote further transmission of the virus (Claudel et al., 2018).

Due to the highly polyphagous nature of *M. persicae,* TuYV can persist in weed species outside of the brassica growing season. However, Newbert (2016) showed that while some weed isolates were able to infect oilseed rape, others were unable to and were genetically distinct from crop isolates. TuYV is a phloem limited virus and relies on aphid vectors for transmission. It cannot be transmitted by contact, mechanically (through wounding) or through the seed like other plant viruses.

### 5.3. Diagnostics

Diagnosis of TuYV infection of brassicas by visual symptoms alone is challenging as they are not always apparent and when present are non-specific and can easily be mistaken for other phenotypes such as nutrient deficiency. Serological techniques such as ELISA using commercially available antibodies can be used to test plant tissue (D’Arcy et al., 1989; Greer et al., 2021a) and molecular techniques such as RT-PCR and LAMP can be used to detect TuYV in plants and aphids (Newbert, 2016; Congdon et al., 2019). Plant material is often not tested for TuYV as once the virus is present in the crop there is little that can be done to control/remove it. It is more informative to test aphids for TuYV, as these results can be fed into forecasting tools. The Department of Primary Industries and Regional Development, Government of Western Australia have developed such a tool that informs growers of aphid and TuYV pressure so that they can tailor their prevention and management practices accordingly (Congdon, 2021).

### 5.4. Prevention and management

#### 5.4.1. Farm practices

Management of aphids is key for the control of TuYV. Monitoring tools can be used to assess aphid activity and help time the drilling and transplanting of brassicas to the field after the peak aphid migrations, where transmission of TuYV will be lower. This also minimizes early infection of the crop which is generally more detrimental than late infection (Congdon et al., 2020). Further, it is also important to remove weeds and manage green-bridges where aphids may find an alternative host and reservoir the virus between crop rotations (AHDB, 2023b). For similar reasons, brassicas should not be grown in fields near other brassica crops or alternative hosts.

#### 5.4.2. Chemical control

Viruses cannot be directly targeted by chemical sprays or seed treatments like other phytopathogens (bacteria and fungi), instead chemicals target the viral vector. Unfortunately, the primary vector of TuYV, *M. persicae* has evolved resistance mechanisms to many of the available insecticide treatments (Bass et al., 2014). Most of the *M. persicae* population possess modified acetylcholinesterase (MACE), knockdown (kdr) or super-kdr resistance that render organophosphates, carbamates and pyrethroid insecticides ineffective (IRAG, 2021).

Insecticides belonging to the pyridine azomethines, sulfoximines and neonicotinoids still remain effective against *M. persicae*, although resistance to the latter has already been reported in Southern mainland Europe, North Africa and Australia (Slater et al., 2012; de Little et al., 2017; Charaabi et al., 2018). To maintain their effectiveness, it is imperative to rotate insecticides belonging to different chemical groups and apply in an informed, and restricted, manner. For example, using seed treatments and foliar sprays early in the growing period, where TuYV infection has its greatest yield impact (Congdon et al., 2020) could be the more effective and more resource efficient than applying treatments throughout the entire growing season. Furthermore, forecasting tools can be used to inform the timings of chemical applications to provide maximum protection, e.g., to coincide with high aphid numbers. It is important to note that some of the effective insecticides may not be approved for use in a given country and pose another hurdle for the control of TuYV. For example, neonicotinoids were banned for use in the EU in 2013 as they were found to be detrimental to pollinator species (The European Commission, 2013).

#### 5.4.3. Biological control

Like chemical controls, biological controls target the aphid vector. There are over 150 natural enemies (pathogen, parasite or predator) of *M. persicae* but most are not used on brassica crops (CABI, 2022c). India uses several predatory species of hoverflies like *Sphaerophoria indiana*, *Ischiodon scutellaris*, *Eupeodes latilunulatus* and *E. confrater* to control *M. persicae* in mustard (*B. nigra* and *B. juncea*). However, these species are only found in India or countries of the Asia-Pacific (CABI, 2022c). Conversely, *Lecanicillium muscarium* and *Beauveria bassiana* are widespread entomopathogenic fungi. They are approved for use in Europe, Asia and America to control whitefly, thrips and aphids like *M. persicae* in vegetable crops (Lewis et al., 2016). Another recent study by Paliwal et al. (2022) identified entomopathogenic bacterial species that killed different clones of *M. persicae* but were non-pathogenic to plants. This is a promising finding but will require further research and development before it could be marketable.

RNA interference (RNAi) has the potential for the control of TuYV. RNAi is a natural gene regulatory mechanism in eukaryotes. It involves the sequence-specific recognition of single- and double-stranded RNA and either tags them for degradation or prevents their translation. The TuYV genome comprises of a single stranded RNA molecule, and it produces double-stranded RNA intermediates during its replication, making it a potential target for RNAi. This has been achieved for other viruses, Lindbo and Dougherty (1992) engineered tobacco plants to express a modified non-functional coat protein of the Tobacco Etch Virus which in turn induced resistance. In a similar way, resistance to *M. persicae* was achieved in tobacco by engineering it to express aphid gene fragments (Feng and Jander, 2022). However, RNAi would require approval as it is a genetic engineering/modification technique.

#### 5.4.4. Resistant cultivars

Host resistance to TuYV is one of the only ways to directly control the virus. Several TuYV resistances have been identified, mainly in *B. napus* (Thomas et al., 1990; Coutts et al., 2010) but only one, the ‘R54’ resistance has been deployed commercially. There are numerous TuYV-resistant oilseed rape varieties but there are no vegetable brassica resistant varieties, although some claim generalized virus resistance (Table 1). The ‘R54’ resistance originated from *B. napus* and was first reported by Graichen (1994). It has subsequently been characterized and mapped to a single dominant QTL on chr. A04 (*B. rapa* A genome) (Dreyer et al., 2001; Juergens et al., 2010). Another resistance from oilseed rape line ‘Yudal’ was mapped to the same genetic locus as the ‘R54’ resistance, but further marker analyses suggested that they may be different in origin (Hackenberg et al., 2020). A study by Congdon et al. (2021) identified additional TuYV resistance source in *B. napus*, *B. oleracea*, and *B. rapa* and showed that at higher temperatures these resistance could breakdown. This is important to consider as some resistances may not be effective in countries with warmer climates or more temperate countries in the face of climate change. Further work is also needed to assess whether the identified resistances are effective against genetically diverse TuYV isolates (Newbert, 2016). Lastly, to ensure the durability of TuYV resistances they should be stacked to reduce the selection pressure for resistance-breaking isolates of TuYV. This was achieved by Greer et al. (2021a) who stacked TuYV resistances from *B. rapa* (genome AA) and *B. oleracea* (genome CC) in *B. napus* (genome AACC).

Another angle for the control of TuYV is the use of *Brassica* cultivars resistant to *M. persicae* but none have been reported. However, resistance to *M. persicae* has been reported in other hosts like peach and the genes responsible *Rm1*, *Rm2*, and *Rm3* (Lambert and Pascal, 2011; Pascal et al., 2017; Niu et al., 2018) have been cloned *and* perhaps could be integrated in *Brassica* crops though genetic modification techniques. However, while some of the resistances are extreme it only takes one aphid and short feeding time of just 15 min to transmit TuYV (Stevens et al., 2008). Thus, host resistance to aphids alone may not provide adequate protection against TuYV in brassica.

## 6. Summary and future perspectives

Black rot (bacterial), clubroot (protist), downy mildew (oomycete) and aphid transmitted turnip yellows virus (aphid and viral) represent vastly different classes of pests and pathogens that cause considerable yield losses on brassicas worldwide. They have complex control strategies where no single preventative or management method discussed in this review is effective on its own. It is therefore important to develop IPMs that combine multiple control methods to reduce pests to tolerable levels with minimal effect on the environment and minimal costs to the pest manager. The five pillars of IPM are (1) identification of pest, host, and beneficial microbes (2) developing monitoring guidelines that are species specific (3) determine action thresholds for the pest (4) evaluate and implement control strategies and finally (5) documenting the results. Although, developing an IPM strategy is a long-term process that requires time, resource, and energy it is the most efficient strategy to control diseases of *Brassica*.

Many of the diagnostics identified in this review are implemented post-disease establishment and by this time, farmers and grower have identified the problem and do not need confirmational diagnostics. Many are also specialized and are lab-based (PCR, ELISA, FAME etc.). Diagnostics will be useful for testing farm-saved seed and identifying prevalent pathotypes/races of a pathogen for resistance cultivar deployment, but they need to be rapid and in-field. Another major limitation of diagnostics is that there is still a huge knowledge gap in pathogen biology/lifecycle, that in turn impedes disease detection and monitoring. Several IOT tool have been developed for disease forecasting, detection, monitoring and management but these are often generalist, e.g., for bacterial disease of brassicas. Specialist host-pathogen specific IOT tools need to be developed.

There are several practices, mainly cultural that are applicable for control of all the diseases discussed in this review (Table 3), but others are disease specific (chemical, biocontrol, host resistance). Cultural controls like seed sterilization and sanitation of field and glasshouse equipment are generally cheap and easy to implement. Removal of weeds could be helpful in some instances, but there is evidence to suggest that weed isolates can be distinct from those infecting crops and there is a balance between crop protection and maintaining biodiversity. Timing of drilling or transplanting of *Brassica* crops may be beneficial to prevent one disease but may exacerbate another. For example, early planting of brassicas reduces clubroot severity, but early sowing and infection increases yield losses to TuYV (Gossen et al., 2012; Congdon et al., 2020).

**Table 3:** Farm practices that can be used to control pests and diseases of brassicas.

Practice	Black rot	Clubroot	Downy mildew	Aphids	TuYV
Seed sterilisation	Y	Y	Maybe	-	-
Sterilisation of glasshouse and farm equipment	Y	Y	Y	-	-
Ebb and flow watering system	Y	Y	Y	-	-
Removal of weeds	Maybe	Y	Y	Y	Maybe
Removal of crop debris and soil sterilization	Y	Y	Y	-	-
Soil liming	?	Y	?	-	-
Crop rotation of 3+ years	Y	Y	Y	?	Y
Early/Late drilling or transplanting	?	Y	?	Y	Y

Y, yes, can be used to control the pest or disease; –, not applicable; ?, further research is required to determine whether it can control the pest or disease.

Chemical controls are damaging to the environment and generally not effective because of evolving resistance mechanisms brought about by over dependence and misuse/overuse of single products. Furthermore, the active chemistry (mode of action) used may not be specific to the pathogen and therefore have limited effectiveness, e.g., fungicides are used to control the clubroot and downy mildew pathogens which are protists and oomycetes, respectively. There are also no chemicals that directly target viruses. Forecasting can help inform precise chemical applications which will in turn maximize their effectiveness, reduce environmental impact and ensure their longevity.

Biocontrols are promising and are generally more precise than chemical applications which can kill beneficial microbiota. However, very few biocontrol agents are currently available for *Brassica* pests and diseases. Most are in the research stages and require considerably more development and time to deliver to market. Some may also meet consumer resistance and will be subject to local laws and regulations due to them being, gene-editing/−modification techniques or non-native species.

Numerous resistance sources to each of the diseases discussed here have been identified, characterized, and mapped in *Brassica* but very few have reached the stage where they have been deployed in the field. A limited resistance base causes strong selection pressure for resistance breaking isolates and so there is an increased risk of resistance break down. Ideally, multiple resistances for each pathogen, pathotype and race would be stacked, or rotated to prevent this. However, resistance often comes with a yield penalty of its own. Introgression of resistance into commercial crop types can take >10 years using traditional breeding methods for even simple single gene resistance. This can be accelerated using gene-editing/−modification techniques, although like biocontrol, this would be subject to local laws and regulations. Rapid in-field diagnostics could help identify pathotypes and races prevalent in the field which would help inform which resistances to deploy and help prevent resistance break down.

Although not discussed in depth in this review, it is also important to consider that disease severity and yield losses caused by disease can be heavily influenced by abiotic factors (weather, nutrient availability, soil pH etc.). For example, calcium and boron levels in the soil play a significant role in clubroot disease management (Saharan et al., 2021). How abiotic factors influence diseases of brassicas and their management is still not clear. Similarly, it is not clear how pathogens interact with other pathogens or beneficial microbes in a field setting as traditional plant pathology is focused on the study of single pathogen-plant host systems. There are still several knowledge gaps that need to be addressed for successful control of the *Brassica* diseases discussed in this review. These are outlined in Table 4 along with our future recommendations for disease prevention and management.

**Table 4:** Knowledge gaps and future recommendations for controlling important diseases of brassicas.

	Black rot	Clubroot	Downy mildew	TuYV
Knowledge gaps	Translation of biocontrols and host resistance research into marketable products/cultivars. Investigation of effect of biocontrols impact beneficial microbiota. Understanding of pathogen diversity, e.g., pathotype and race structure and their geographical prevalence. Identification of broad-spectrum resistances, e.g., to multiple pathotypes and races. Lack of understanding of pathogen life cycles at the molecular and biochemical levels. Lack of early diagnostics before symptom development. Investigating how abiotic factors, microbiota and co-infection with different pathogens affect disease development and severity.			
Future recommendations	Sowing certified *Xcc*-free seed. Use of hot water and hydrogen peroxide seed sterilisation. Use ebb and flow glasshouse watering systems and treatment of irrigation water with chlorine dioxide. Use of bacteriocins and phages for disease control. Use and rotate cultivars with resistances to the prevalent races in the area.	Development of axenic culture for *P. brassicae* could make it easier to study its life cycle and identifying the stages to target with control methods. Use of GPS and GPI to survey land for clubroot prior to planting. Use of disease suppressive soils and soil solarisation. Use of bio stimulants and biocontrol agents like Serenade^®^ and Mycostop^®^ for disease control. Development and use of new resistance cultivars other than Mendel and Tosca.	Development of axenic culture for *H. parasitica* could make it easier to study its life cycle and identifying the stages to target with control methods. Use ebb and flow glasshouse watering systems. Use of metalaxyl in combination with biochemicals and biocontrol agents (*Trichoderma* spp.) that stimulate systemic acquired resistance. Development and use of resistance cultivars with durable resistance resilient to rapid *H. parasitca* evolution because of its sexual cycle.	Use forecasting tools to monitor vector numbers and migrations to inform drilling and transplanting date. Manage weed species and green bridges to eliminate reservoirs for the virus outside of crop growing season. Use predatory insect or entomopathogenic microbes to control virus vectors. Use RNAi to directly target TuYV and/or its aphid vector. Development and use of new resistance cultivars to minimize risk of resistance break down in current cultivars.
	Development of an IPM strategy (cultural, chemical, biocontrol and host resistance) to reduce chemical input and environmental impact, maintain biodiversity and ensure durable disease control. Tailoring of management strategies to specific crops and geographical regions. Precision targeting of phytopathogens with host resistance, biocontrol agents and gene-editing/−modification techniques, e.g., RNAi. Development of AI for disease forecasting detection, monitoring, and management. Develop farmer friendly forecasting and management tools to help inform them of the most effective control agents and application timings.			

## Author contributions

SG and AS: conceptualization, methodology, and writing—original draft. MG and RL: supervision, validation, and writing—reviewing and editing. All authors contributed to the article and approved the submitted version.

## Funding

This work was funded by a grant from BBSRC, NERC, Defra, and the Scottish Government, under the Strategic Priorities Fund Plant Bacterial Diseases program under the project ‘Xanthomonas plants diseases: mitigating existing, emerging and future threats to UK agriculture’ (BB/T010924/1) and a grant from Innovate UK under the project ‘Insectrial Revolution’ (ISCF FFPS 47278). The Open Access publication costs were provided by the UKRI block grant to the University of Warwick.

## Conflict of interest

The authors declare that the research was conducted in the absence of any commercial or financial relationships that could be construed as a potential conflict of interest.

## Publisher’s note

All claims expressed in this article are solely those of the authors and do not necessarily represent those of their affiliated organizations, or those of the publisher, the editors and the reviewers. Any product that may be evaluated in this article, or claim that may be made by its manufacturer, is not guaranteed or endorsed by the publisher.
